# Real-world effectiveness of sotrovimab in preventing hospitalization and mortality in high-risk patients with COVID-19 in the United States: A cohort study from the Mayo Clinic electronic health records

**DOI:** 10.1371/journal.pone.0304822

**Published:** 2024-07-16

**Authors:** Christopher F. Bell, Daniel C. Gibbons, Myriam Drysdale, Helen J. Birch, Emily J. Lloyd, Vishal Patel, Corinne Carpenter, Katherine Carlson, Ediz S. Calay, Arjun Puranik, Tyler E. Wagner, John C. O’Horo, Raymund R. Razonable

**Affiliations:** 1 GSK, Durham, North Carolina, United States of America; 2 GSK, Brentford, United Kingdom; 3 nference, Cambridge, Massachusetts, United States of America; 4 Mayo Clinic, Rochester, Minnesota, United States of America; Faculty of Pharmacy - Istinye University, TURKEY

## Abstract

**Background:**

To describe outcomes of high-risk patients with coronavirus disease 2019 (COVID-19) treated with sotrovimab, other monoclonal antibodies (mAbs), or antivirals, and patients who did not receive early COVID-19 treatment. We also evaluate the comparative effectiveness of sotrovimab versus no treatment in preventing severe clinical outcomes.

**Methods:**

This observational retrospective cohort study analyzed Mayo Clinic electronic health records. Non-hospitalized adult patients diagnosed with COVID-19 from May 26, 2021 and April 23, 2022 and at high risk of COVID-19 progression were eligible. The primary outcome was 29-day all-cause hospitalization and/or death. Outcomes were described for patients treated with sotrovimab, other mAbs, or antivirals, and eligible but untreated patients, and compared between sotrovimab-treated and propensity score (PS)-matched untreated cohorts.

**Results:**

We included 35,485 patients (sotrovimab, 1369; other mAbs, 6488; antivirals, 133; high-risk untreated, 27,495). A low proportion of patients treated with sotrovimab (n = 33/1369, 2.4%), other mAbs (n = 147/6488, 2.3%), or antivirals (n = 2/133, 1.5%) experienced all-cause hospitalization or death. Among high-risk untreated patients, the percentage of all-cause hospitalization or death was 3.3% (n = 910/27,495). In the PS-matched analysis, 2.5% (n = 21/854) of sotrovimab-treated patients experienced all-cause hospitalization and/or death versus 2.8% (n = 48/1708) of untreated patients (difference, –0.4%; p = 0.66). Significantly fewer sotrovimab-treated patients required intensive care unit admission (0.5% vs 1.8%; difference, –1.3%; p = 0.002) or respiratory support (3.5% vs 8.7%; difference, –5.2%; p < 0.001).

**Conclusions:**

There was no significant difference in the proportion of sotrovimab-treated and PS-matched untreated patients experiencing 29-day all-cause hospitalization or mortality, although significantly fewer sotrovimab-treated patients required intensive care unit admission or respiratory support.

## Introduction

Globally, as of July 2023, there have been over 760 million confirmed cases of coronavirus disease 2019 (COVID-19), and nearly 7 million deaths [1]. In the US, over 1.1 million deaths have been attributed to COVID-19 over the same period [1]. Patients at highest risk of poor clinical outcomes include individuals aged ≥ 65 years; those with obesity, diabetes mellitus, hypertension, advanced renal and liver disease, or chronic lung and cardiac conditions; immunocompromised patients; and those with multiple comorbidities [2–5].

Before November 2020, there were no approved treatments to prevent progression to severe COVID-19. The US Food and Drug Administration (FDA) has since granted Emergency Use Authorization (EUA) for several neutralizing anti-spike monoclonal antibodies (mAbs) and antiviral drugs for early outpatient treatment of mild-to-moderate COVID-19 to prevent clinical progression, hospitalization, and death [6], including sotrovimab in May 2021 [7]. Following the emergence of the Omicron BA.2 variant, the US Food and Drug Administration deauthorized sotrovimab on a state-by-state basis from March 2022, with a national deauthorization occurring on April 5, 2022 [8]. Sotrovimab remains authorized in other countries, including the UK [9], and within the EU [10].

The withdrawal of EUAs for mAbs in the US was based, in part, on the results of in vitro neutralization experiments for variants of concern. At the time of study initiation, it was unclear if and how these laboratory results translated to changes in clinical effectiveness, although there is now an improved understanding based on real-world evidence [11–15]. Insights from real-world early use of mAbs for COVID-19 complement clinical trial data and help to assess the impact of changes to in vitro neutralization potency of mAbs on their clinical effectiveness against corresponding viral variants. There is a need to generate high-quality real-world data on the clinical effectiveness of mAbs considering the changing COVID-19 environment (e.g., variants, subpopulations, vaccination status, and public policies), and to understand effectiveness among real-world populations rather than highly selective clinical trial cohorts.

Here, we describe real-world use and outcomes data from high-risk patients treated with sotrovimab, other mAbs, and antivirals, as well as untreated patients, who were diagnosed with COVID-19 at Mayo Clinic between May 26, 2021 (sotrovimab authorization) and April 23, 2022 (last recorded administration of sotrovimab at Mayo Clinic), when the predominant circulating variants of concern were Delta, Omicron BA.1, and Omicron BA.2 [16, 17]. We also assessed the comparative effectiveness of sotrovimab versus no treatment among propensity score (PS)-matched patients.

## Methods

### Study design and data source

This observational retrospective cohort study used electronic health record (EHR) data from patients at Mayo Clinic, an integrated healthcare delivery network serving more than one million patients annually across Minnesota, Florida, Arizona, Iowa, and Wisconsin. The network employs a single EHR system (Epic Systems Corporation), allowing all sites across the network to be connected.

On November 7, 2020, Mayo Clinic established its Monoclonal Antibody Treatment (MATRx) Program to administer mAbs (and subsequently antivirals) to high-risk patients with mild-to-moderate COVID-19. The program created COVID-19-dedicated multidisciplinary teams and infusion units in anticipation of the US FDA EUA of mAbs for COVID-19. The MATRx Program, protocols, and procedures have been described previously [18,19], as have real-world outcomes of patients receiving early treatment (mAb and antiviral) at Mayo Clinic [20–24].

Patient eligibility for treatment was reviewed by the MATRx team according to the US FDA EUA criteria (S1 Table in S1 File) using both EHR tools for internal patients, and a self- and clinician-referred process for patients external to the health system. To assist in the identification of patients who were at an elevated risk of hospitalization and who would benefit the most from mAb treatment, the Monoclonal Antibody Selection Score (MASS) was developed and was subsequently updated in May 2021 to adapt the expanded treatment for eligibility criteria (and re-named the COVID Antibody Screening Tool Score [CAST]) [25]. Both MASS and CAST leveraged the Mayo Clinic EHR system to evaluate the US FDA EUA eligibility criteria, where patients were eligible for treatment if they had a positive SARS-CoV-2 PCR or antigen test, mild-to-moderate COVID-19, were within 10 days of symptom onset, and had at least one of the following criteria: age ≥65 years, BMI ≥35 kg/m^2^, diabetes mellitus, chronic kidney disease, immunosuppressive medication use, or an immunocompromising condition. Patients aged ≥55 years also qualified if they had hypertension, cardiovascular disease, or chronic lung disease [25].

MASS and CAST were originally intended to risk-stratify patients to guide the allocation of scarce mAb resources (limited supply, limited capacity, or staff shortages); however, the availability of mAbs improved and the tools were subsequently used to identify patients who were eligible for mAb treatment [21, 25, 26]. If eligible, patients received information and education about treatment options, the potential benefits and adverse effects, and the EUA status of all products. Treatment decisions were based on patient factors (e.g., comorbidities, drug-drug interactions), drug factors (e.g., mAbs were distributed to infusion facilities by the Federal government with the choice of specific mAb based on availability at the facility on the day of treatment), and patient preference after shared decision-making between patients and providers. All patients provided consent for treatment, with consenting patients immediately scheduled for infusion at any Mayo Clinic site. All treatments were administered under the EUA guidance. Patients were monitored prior to, during, and for one hour after infusions, and were subsequently followed by a remote monitoring program [18, 25].

This study was reviewed by the Mayo Clinic Institutional Review Board (IRB) and granted exempt status (Mayo Clinic IRB 20–012919). Only patients with research authorization on file were included in the current study.

### Study population

This observational retrospective cohort study was conducted among patients identified from the Mayo Clinic EHR database. The cohort consisted of adults (age ≥18 years) diagnosed with COVID-19 between May 26, 2021 and April 5, 2022, which (per the MATRx Program employed across Mayo Clinic Health sites) included patients with a positive SARS-CoV-2 test or COVID-19 diagnosis (ICD-10: U07.1). The start and end dates were selected to reflect the EUA period for sotrovimab.

The cohort was divided based on the treatment received during the study period. Patients eligible for treatment with a mAb or an antiviral (i.e., mild-to-moderate COVID-19, symptom onset within 10 days [mAb treatment] or 5 days [antiviral treatment], meeting FDA EUA criteria) were assigned to one of the following treatment cohorts based on the first outpatient or emergency department treatment received during the study period: sotrovimab, other mAb (bamlanivimab, bamlanivimab-etesevimab, casirivimab-imdevimab, betelovimab), or antiviral drug (nirmatrelvir/ritonavir, molnupiravir). Patients may have been administered medications within other categories following initiation of the first medication (e.g., patients who were administered sotrovimab and subsequently administered an antiviral drug [if they developed severe disease progression and required subsequent hospitalization] were included in the sotrovimab cohort). Eligible patients who did not receive treatment were assigned to a control cohort (i.e., untreated). All eligible patients were also required to have sufficient data during the 29-day follow-up period to determine vital status, defined as either complete follow-up beyond Day 29, or death occurring within the follow-up period. Reasons for insufficient data include changes in a patient’s healthcare provider, or an out-of-state patient who received care within the Mayo Clinic system.

Patients were excluded from the treatment or control cohorts if they were previously (in the past 12 months) administered a mAb, antiviral or tixagevimab/cilgavimab for the treatment or prevention of COVID-19 progression, or if they were hospitalized in the 14 days prior to treatment (14 days prior to a positive test or COVID-19 diagnosis in the control group). Patients were also excluded from the control cohort if they had been administered sotrovimab, another mAb, or an antiviral (nirmatrelvir-ritonavir or molnupiravir) ≥10 days after COVID-19 diagnosis.

### Study outcomes

Study variables included patient demographics (e.g., age, gender, race/ethnicity, geographic region), patient characteristics (e.g., smoking history, weight, BMI), medical comorbidities (e.g., EUA high-risk criteria, Charlson-comorbidity index, MASS), and COVID-19 testing, vaccination and treatments. Clinical outcomes assessed for the 29-day follow-up period were hospitalization, death, ICU admission, and respiratory support (oxygen therapy, non-invasive ventilation, invasive mechanical ventilation, extracorporeal membrane oxygenation). The primary outcome of interest was a composite measure of all-cause hospitalization and/or all-cause mortality in the 29-day follow-up period.

### Data analysis

Descriptive summary statistics were calculated for all study variables and clinical outcomes across the three treatment cohorts (sotrovimab, other mAb, antiviral) and the control (i.e., untreated) cohort. Continuous variables (e.g., age) were summarised using mean and standard deviation (SD). Categorical variables (e.g., gender) were described using frequencies and percentages. No inferential statistics were performed for these cohorts. For each patient in the treated cohorts, the index date was defined as the date of treatment (i.e., infusion for the mAbs and prescription for antivirals). For each patient in the control cohort, the index date was defined as the date of positive test or COVID-19 diagnosis since there was no treatment date. While this does create different reference periods, it is important to note that this assessment was descriptive so as to provide contextualization of the outcomes of interest. Furthermore, data from previous studies have shown that the median time to mAb infusion was 2 days after diagnosis of COVID-19 [20, 23]. Finally, observations from this descriptive analysis indicated that approximately 90% of patients received their mAb infusion within 3 days of COVID-19 diagnosis with <3% of infusions occurring after day 7.

Propensity-score matching was employed to construct 1:2 matched cohorts of sotrovimab-treated and control (i.e., untreated) patients with similar clinical characteristics. A logistic regression model was used to calculate the propensity score for each patient based on the following covariates: demographics (sex, race, ethnicity; S2 Table in S1 File); clinical characteristics and comorbidities (S3 Table in S1 File); and vaccination status (S4 Table in S1 File). Patients in the control cohort were matched to patients in the sotrovimab-treated cohort based upon the following exact matching requirements: age (+/- 5 years), COVID-19 testing date (+/- 14 days), state of residence, and the corresponding propensity scores. Control patients were assigned a putative treatment date based on their COVID-19 test date and the difference between their match’s COVID-19 test date and sotrovimab infusion date, with control patients who were hospitalized between their COVID-19 test and putative treatment date excluded from the analysis. Matching was done without replacement using greedy nearest-neighbor matching with a caliper of 0.2 * the pooled standard deviation of scores. The effectiveness of propensity score matching in covariate balancing between the sotrovimab-treated and control cohorts was assessed using standardized mean differences, with a value ≤0.10 considered representative of adequate balance between cohorts.

Clinical outcomes, including the primary outcome of interest (composite measure of all-cause hospitalization and/or all-cause mortality in the 29-day follow-up period), were compared across the sotrovimab-treated and control cohorts using the appropriate statistical test (e.g., Fisher exact test, Mann-Whitney U test). Results are reported in terms of standard summary statistics (numbers, percentages, means, standard deviations) and absolute differences with associated 95% confidence internals. Statistical significance was evaluated at the p<0.05 level.

## Results

### Patient identification

A total of 210,931 patients in Mayo Clinic EHR database had a COVID-19 diagnosis by a positive SARS-CoV-2 PCR test or ICD-10 diagnosis code U07.1. Following application of the eligibility criteria, the final analysis included 1369 patients treated with sotrovimab, 6488 patients treated with other mAbs, 133 patients treated with antivirals, and 27,495 eligible high-risk untreated patients (Table 1).

**Table 1:** Number of patients identified and included for analysis per cohort.

Inclusion criteria	Sotrovimab N (%)	Other mAbs^a^ N (%)	Antivirals^b^ N (%)	Untreated with mAbs or antivirals N (%)
Received treatment of interest as first treatment in an outpatient or ER setting of care within 10 days of the index date (defined as the earliest of a positive test for SARS-CoV-2 or the date of COVID-19 diagnosis [ICD-10 diagnosis code U07])^c^	1941 (100.0)	8266 (100.0)	165 (100.0)	37,988 (100.0)
Had 29 days of follow-up or died within 29 days of reference date^d^	1378 (80.0)	6534 (79.1)	134 (81.2)	29,152 (76.7)
Not hospitalized within 14 days before reference date^d^	1369 (70.5)	6489 (78.5)	133 (80.6)	27,495 (72.4)
No medications of interest within 1 year before reference date^e^	1369 (70.5)	6488 (78.5)	133 (80.6)	27,495 (72.4)
Final number included for analysis in each cohort	1369	6488	133	27,495

^a^Treatment of interest in the other mAbs cohort included bamlanivimab, bamlanivimab-etesevimab, casirivimab-imdevimab, and bebtelovimab.

^b^Treatment of interest in the antivirals cohort included nirmatrelvir-ritonavir and molnupiravir.

^c^Patients not receiving any treatment of interest within 10 days of the index date were assigned to the untreated (control) cohort.

^d^Reference date is the treatment date for the sotrovimab, other mAbs, and antivirals cohorts, and the date of positive test for SARS-CoV-2 or COVID-19 diagnosis for the untreated cohort.

^e^Patients should not have previously been administered a mAb (e.g., sotrovimab, bamlanivimab, bamlanivimab-etesevimab, casirivimab-imdevimab, bebtelovimab), antiviral (nirmatrelvir-ritonavir, molnupiravir, or remdesivir), or tixagevimab-cilgavimab within the past 12 months.

COVID-19, coronavirus disease 2019; ER, emergency room; ICD-10, International Classification of Disease version 10; mAb, monoclonal antibody; SARS-CoV-2, severe acute respiratory syndrome coronavirus 2.

### Patient demographics and clinical characteristics (unmatched)

The mean (SD) age of the untreated cohort was 53.2 (18.5) years; this was 59.5 (17.5), 59.0 (16.8), and 65.3 (15.0) years in the sotrovimab-, other mAb-, and antiviral-treated cohorts, respectively (Table 2). Across the treated cohorts, most patients were female (51.9–58.1%), Caucasian (90.6–94.6%), and had a mean body mass index ≥ 30 kg/m^2^ (34.4–36.0 kg/m^2^).

**Table 2:** Patient demographics and clinical characteristics across the unmatched cohorts.

	Sotrovimab N = 1369	Other mAbs^a^ N = 6488	Antivirals^b^ N = 133	Untreated with mAbs or antivirals N = 27,495
Age (years), mean (SD)	59.5 (17.5)	59.0 (16.8)	65.3 (15.0)	53.2 (18.5)
Female, n (%)	794 (58.0)	3672 (56.6)	69 (51.9)	15,965 (58.1)
Caucasian, n (%)	1273 (93.0)	6140 (94.6)	121 (91.0)	24,903 (90.6)
Body mass index (kg/m^2^), mean (SD)	34.4 (8.8)	35.0 (8.7)	36.0 (9.2)	34.8 (8.6)
Mayo Clinic site location, n (%)				
Rochester, Minnesota	413 (30.2)	1217 (18.8)	59 (44.4)	5754 (20.9)
Jacksonville, Florida	128 (9.4)	713 (11.0)	3 (2.3)	3231 (11.8)
Scottsdale, Arizona	153 (11.2)	761 (11.7)	6 (4.5)	3525 (12.8)
Other MCHS locations, Minnesota, and Wisconsin	675 (49.3)	3795 (58.5)	65 (48.9)	14,970 (54.5)
COVID-19 vaccination status, n (%)				
No record	356 (26.0)	2412 (37.2)	18 (13.5)	11,638 (42.3)
Partial^c^	68 (5.0)	290 (4.5)	4 (3.0)	1271 (4.6)
Fully^d^	435 (31.8)	3387 (52.2)	39 (29.3)	9506 (34.6)
Boosted^e^	510 (37.3)	399 (6.2)	72 (54.1)	5080 (18.5)
Smoking status, n (%)				
No record	764 (55.8)	3472 (53.5)	80 (60.2)	16,241 (59.1)
Confirmed non-smoker	369 (27.0)	1795 (27.7)	30 (22.6)	6541 (23.8)
Former or current smoker	236 (17.2)	1221 (18.8)	23 (17.3)	4706 (17.1)
Alcohol use, n (%)				
No record	767 (56.0)	3483 (53.7)	80 (60.2)	16,286 (59.3)
Confirmed non-drinker	165 (12.1)	741 (11.4)	12 (9.0)	2747 (10.0)
Former or current drinker	437 (31.9)	2263 (34.9)	41 (30.8)	8456 (30.8)
Quan-Charlson Comorbidity Index, mean (SD)	2.5 (2.2)	2.2 (2.0)	2.5 (1.7)	1.65 (1.9)
Quan-Charlson Comorbidity Index, n (%)				
0	316 (23.1)	1677 (25.9)	20 (15.0)	10,822 (39.4)
1	165 (12.1)	1018 (15.7)	20 (15.0)	4449 (16.2)
2	258 (18.0)	1288 (19.9)	30 (22.6)	4508 (16.4)
≥ 3	630 (46.0)	2505 (38.6)	63 (47.4)	7716 (28.1)
mAb screening score^f^, mean (SD)	3.8 (3.0)	3.2 (2.8)	3.4 (2.4)	2.4 (2.5)
EUA criteria, n (%)				
Aged ≥ 65 years	658 (48.1)	2800 (43.2)	78 (58.6)	8796 (32.0)
Cardiovascular disease	253 (18.5)	1134 (17.5)	19 (14.3)	3233 (11.8)
History of CKD (any stage)	114 (8.3)	433 (6.7)	5 (3.8)	1202 (4.4)
History of CKD (stage ≥ 3)	50 (3.7)	164 (2.5)	1 (0.8)	448 (1.6)
History of type 2 diabetes	177 (12.9)	799 (12.3)	20 (15.0)	2203 (8.0)
History of type 1 diabetes	15 (1.1)	52 (0.8)	0 (0)	196 (0.7)
Chronic lung disease	90 (6.6)	518 (8.0)	7 (5.3)	1700 (6.2)
Liver disease	60 (4.4)	192 (3.0)	4 (3.0)	715 (2.6)
Sickle cell disease	0 (0)	1 (0)	0 (0)	11 (0)
Neurodevelopmental disorders	18 (1.3)	128 (2.0)	2 (1.5)	798 (2.9)
Pregnancy	159 (11.6)	415 (6.4)	1 (0.8)	2474 (9.0)
Obesity	885 (64.6)	4505 (69.4)	98 (73.7)	19,396 (70.5)
Medical-related technology dependence^g^	0 (0)	0 (0)	0 (0)	3 (0)
Immunocompromised	468 (34.2)	1167 (18.0)	12 (9.0)	4171 (15.2)
Solid organ transplant	213 (15.6)	520 (8.0)	5 (3.8)	1755 (6.4)
SCT/BMT transplant in past year	52 (3.8)	82 (1.3)	0 (0)	284 (1.0)
Anti-CD20 medication in past year	39 (2.8)	72 (1.1)	0 (0)	195 (0.7)
Tier 1 immunocompromised^h^	281 (20.5)	634 (9.8)	5 (3.8)	2112 (7.7)
Immunosuppressive disease	162 (11.8)	498 (7.7)	8 (6.0)	1749 (6.2)
Immunosuppressive treatment^i^	312 (22.8)	486 (7.5)	2 (1.5)	1709 (6.2)

^a^Treatment of interest in the other mAbs cohort included bamlanivimab, bamlanivimab-etesevimab, casirivimab-imdevimab, and bebtelovimab.

^b^Treatment of interest in the antivirals cohort included nirmatrelvir-ritonavir and molnupiravir.

^c^Patients were considered partially vaccinated if they had received 1 vaccine during assessment period with an mRNA vaccine (Pfizer-BioNTech [BNT162b2] or Moderna [mRNA-1273]).

^d^Patients were considered fully vaccinated if they had received ≥ 2 vaccinations during the assessment period with an mRNA vaccine (Pfizer-BioNTech [BNT162b2] or Moderna [mRNA-1273]) or had received a single dose of viral vector Johnson & Johnson vaccine (JNJ-784336725).

^e^Patients were considered fully vaccinated with a booster if they had received ≥ 3 vaccinations with an mRNA vaccine, or ≥ 2 vaccinations, with ≥ 1 of those vaccinations being with JNJ-784336725.

^f^Used by Mayo Clinic to determine treatment eligibility [25].

^g^Defined as the presence of a procedure occurrence or device exposure event relating to any of the following: respiratory aspirator, gastro or jejunostomy, mitrofanoff, a nasogastric tube, renal replacement therapy, total parenteral nutrition, or ventricular assistance.

^h^Tier 1 immunocompromised includes: solid organ transplant recipients (any time frame prior to reference date); recipients of anti-CD20 medication (rituximab, ocrelizumab, ofatumumab, ublituximab) within the past 12 months (including for solid organ transplant); B-cell malignancies, on active treatment (e.g., B-cell lymphomas, chronic lymphocytic leukemia, acute B-cell lymphoblastic leukemia, etc.); allogeneic stem cell transplant, or BMT within prior 12 months.

^i^Defined as ≥2 drug exposure events for systemic corticosteroid therapy in past 365 days or ≥ 1 systemic non-corticosteroid immunosuppressants drug exposures in 365 days.

BMT, bone marrow transplant; COVID-19, coronavirus disease 2019; CKD, chronic kidney disease; EUA, Emergency Use Authorization; mAb, monoclonal antibody; MCHS, Mayo Clinic health system; mRNA, messenger ribonucleic acid; SCT, stem cell transplant; SD, standard deviation.

The proportion of patients considered fully vaccinated against SARS-CoV-2 (including those with and without a booster) varied across the treated cohorts: 69.0% (n = 945/1369) of sotrovimab-treated patients, 58.4% (n = 3786/6488) of other mAb-treated patients, 83.5% (n = 111/133) of antiviral-treated patients, and 53.0% (n = 14,586/27,495) of untreated patients.

Across the cohorts, the most common EUA criterion associated with high risk of COVID-19 progression was obesity (sotrovimab: 64.6%, n = 885/1369; other mAbs: 69.4%, n = 4505/6488; antivirals: 73.7%, n = 98/133; no treatment: 70.5%, n = 19,396/27,495; Table 2). Patients treated with sotrovimab and other mAbs met similar EUA criteria, except for immunocompromised status (sotrovimab: 34.2%, n = 468/1369; other mAbs: 18.0%, n = 1167/6488) and pregnancy (sotrovimab: 11.6%, n = 159/1369; other mAbs: 6.4%, n = 415/6488). In the sotrovimab, other mAbs, antivirals, and untreated cohorts, 48.1% (n = 658/1369), 43.2% (n = 2800/6488), 58.6% (n = 78/133), and 32.0% (n = 8796/27,495) were aged ≥ 65 years, respectively.

### Treatment characteristics (unmatched)

Among the sotrovimab, other mAbs, and antiviral treatment cohorts, there were differences in the month treatment was received (Table 3), reflecting the various treatment authorizations and deauthorizations over the study period. Most patients in the sotrovimab-treated (79.5%, n = 1089/1369), other mAb-treated (76.4%, n = 4955/6488), and antiviral-treated cohorts (94.7%, n = 126/133) received treatment within ≤ 2 days of COVID-19 diagnosis.

**Table 3:** Treatment characteristics across the unmatched cohorts.

	Sotrovimab N = 1369	Other mAbs^a^ N = 6488	Antivirals^b^ N = 133	Untreated with mAbs or antivirals^c^ N = 27,495
Days from most recent vaccination to index, mean (SD)	163.0 (90.3)	164.8 (68.1)	182.0 (96.4)	170.6 (97.5)
Month of treatment, n (%)				
May-21	-	4 (0.1)	-	42 (0.2)
Jun-21	-	64 (1.0)	-	189 (0.7)
Jul-21	-	340 (5.2)	-	593 (2.2)
Aug-21	-	1341 (20.7)	-	1516 (5.5)
Sep-21	-	1577 (24.3)	-	1616 (5.9)
Oct-21	82 (6.0)	1163 (17.9)	-	1662 (6.0)
Nov-21	192 (14.0)	990 (15.3)	-	2605 (9.5)
Dec-21	214 (15.6)	706 (10.9)	-	3459 (12.6)
Jan-22	505 (36.9)	1 (0)	31 (23.3)	12,251 (44.6)
Feb-22	264 (19.3)	-	47 (35.3)	2368 (8.6)
Mar-22	111 (8.1)	62 (1.0)	16 (12.0)	647 (2.4)
Apr-22	1 (0.1)	240 (3.7)	39 (29.3)	547 (2.0)
May-22	-	-	-	-
Days from index to treatment, mean (SD)	1.8 (1.2)	2.0 (1.4)	1.3 (0.7)	-
Days from index to treatment, n (%)				
0	75 (5.5)	229 (3.5)	9 (6.8)	-
1	604 (44.1)	2607 (40.2)	91 (68.4)	-
2	410 (30.0)	2119 (32.7)	26 (19.5)	-
3	166 (12.1)	841 (13)	4 (3.0)	-
≥ 4	114 (8.3)	692 (10.7)	3 (2.3)	-

^a^Treatment of interest in the other mAbs cohort included bamlanivimab, bamlanivimab-etesevimab, casirivimab-imdevimab, and bebtelovimab.

^b^Treatment of interest in the antivirals cohort included nirmatrelvir-ritonavir and molnupiravir.

^c^“Treatment” for the untreated cohort is the date of positive test for SARS-CoV-2 or COVID-19 diagnosis (ICD-10 diagnosis code U07).

COVID-19, coronavirus disease 2019; ICD-10, International Classification of Disease version 10; mAb, monoclonal antibody; SARS-CoV-2, severe acute respiratory syndrome coronavirus 2; SD, standard deviation.

### PS-matched sotrovimab-treated and untreated cohorts

#### Cohort characteristics

In total, 854 sotrovimab-treated patients were PS-matched with 1708 untreated (control) patients; patient characteristics before and after matching, along with SMDs, are summarized in Supplementary Tables 2–4. After PS matching, the sotrovimab and untreated cohorts were well balanced (SMD, ≤ 0.10), except for the following covariates: White/Caucasian race, immunosuppressive treatment, received anti-CD20 medication, and treatment month: December 2021 and January 2022.

**Table 4:** All-cause primary and secondary outcomes across PS-matched sotrovimab-treated and untreated (control) cohorts (29 days from treatment date).

	Sotrovimab N = 854	Control N = 1708	Difference (95% CI)	p-value
Hospitalization or mortality, n (%)	21 (2.46)	48 (2.81)	–0.4% (–1.6%, 1.0%)	0.66
Hospitalization, n (%)	18 (2.11)	30 (1.76)	0.4% (–0.8%, 1.6%)	0.50
Time to first hospitalization, mean days (SD)	12.11 (7.21)	13.03 (8.41)	N/A	N/A
Mortality, n (%)	5 (0.59)	20 (1.17)	–0.6% (–1.3%, 0.2%)	0.18
Time to death, mean days (SD)	17.20 (7.40)	13.10 (7.32)	N/A	N/A
ICU admission, n (%)	4 (0.47)	31 (1.81)	–1.3% (–2.1%, –0.5%)	0.002
Time to first ICU visit, mean days (SD)	9.75 (6.99)	8.00 (8.24)	N/A	N/A
Any respiratory support, n (%)	30 (3.51)	149 (8.72)	–5.2% (–7.0%, –3.3%)	< .001
Days on respiratory support, mean (SD)	0.17 (1.59)	0.38 (1.99)	–0.21% (–0.35%, –0.06%)	0.005

CI, confidence interval; ICU, intensive care unit; N/A, not applicable; PS, propensity score; SD, standard deviation.

#### All-cause (29-day) outcomes across the PS-matched cohorts

All-cause outcomes across the sotrovimab and PS-matched untreated cohorts in the 29-day follow-up period are shown in Table 4. In total, 21 of 854 patients in the sotrovimab cohort were hospitalized or died compared with 48 of 1708 patients in the untreated cohort (2.5% vs 2.8%; difference, –0.4%; 95% CI [–1.6%, 1.0%]; p = 0.66).

In total, 18 of 854 patients in the sotrovimab cohort were hospitalized compared with 30 of 1708 patients in the untreated cohort (2.1% vs 1.8%; difference, 0.4%; 95% CI [–0.8%, 1.6%]; p = 0.50). The mean (SD) time to first all-cause hospitalization was 12.1 (7.2) days in the sotrovimab cohort and 13.0 (8.4) days in the untreated cohort.

In the sotrovimab cohort, 5 of 854 patients died compared with 20 of 1708 patients in the untreated cohort (0.6% vs 1.2%; difference, –0.6%; 95% CI [–1.3%, 0.2%]; p = 0.18). Among patients who died, the mean (SD) time to death was 17.2 (7.4) days in the sotrovimab cohort and 13.1 (7.3) days in the untreated cohort.

Significantly fewer sotrovimab-treated patients (n = 4/854) were admitted to ICU within 29 days compared with untreated patients (n = 31/1708; 0.5% vs 1.8%; difference, –1.3%; 95% CI [–2.1%, –0.5%]; p = 0.002; Table 4).

Significantly fewer sotrovimab-treated patients (n = 30/854) required respiratory support within 29 days compared with untreated patients (n = 149/1708; 3.5% vs 8.7%; difference, –5.2%; 95% CI [–7.0%, –3.3%]; p < 0.001; Table 4).

## Discussion

This study outlines the characteristics and outcomes of high-risk patients who received early treatment for COVID-19 at Mayo Clinic, or those who were eligible but did not receive early COVID-19 treatment for any reason, between May 26, 2021 and April 23, 2022. Although there were lower rates of all-cause hospitalization and death among treated versus untreated high-risk patients, a significant difference was not observed for sotrovimab after PS matching. However, analysis of the sotrovimab-treated and PS-matched untreated cohorts showed that sotrovimab treatment was associated with significantly fewer ICU admissions and significantly less requirement for respiratory support.

The proportion of sotrovimab-treated patients who experienced all-cause hospitalizations and/or death in this study (2.5% in the PS-matched analysis; 2.4% in the unmatched analysis) is consistent with other studies. Of interest, hospitalizations and/or deaths experienced by untreated patients (2.8% in the PS-matched analysis; 3.3% in the unmatched analysis) were lower than has been previously reported. In the US, Cheng et al. evaluated 30-day hospitalization or mortality from September 2021–April 2022 among sotrovimab-treated and PS-matched untreated patients [11]. Among sotrovimab-treated patients, 2.68% (n = 419/15,633) experienced all-cause hospitalization or mortality versus 5.59% (n = 84,720/1,514,868) of untreated patients [11]. Two further studies used EHR data from Colorado to evaluate the effectiveness of sotrovimab compared with a PS-matched cohort of untreated patients for 28-day hospitalization [27, 28]. In the first study (conducted from October 2021–December 2021 when the Delta variant predominated), 2.1% (n = 11/522) of sotrovimab-treated patients were hospitalized compared with 5.7% (n = 89/1563) of the untreated cohort [27]. In the second (conducted from December 2021–March 2022 when Omicron predominated), 2.5% (n = 39/1542) of sotrovimab-treated patients were hospitalized, compared with 3.2% (n = 116/3663) of the untreated cohort [28]. It is possible that the MATRx Program employed by the Mayo Clinic, in which high-risk patients with a positive COVID-19 test were proactively monitored and managed remotely, may have impacted hospitalizations and/or deaths in the control cohort, The remote monitoring and management program could have prevented some patients from seeking hospitalization as they were already receiving care through telemedicine.

Two real-world studies from Mayo Clinic also reported low proportions of patients who developed severe COVID-19 outcomes within 30 days of early treatment with sotrovimab and other mAbs, although these studies did not have an untreated comparator group [18, 29]. In the first study, conducted from August 2021–December 2021, 2.7% (n = 287/10,775) of the overall cohort and 1.6% (n = 17/1072) of sotrovimab-treated cohort progressed to severe COVID-19 and were hospitalized [29]. In the second study, conducted from January 2022–March 2022, 2.2% (n = 49/2182) of sotrovimab-treated patients progressed to severe COVID-19 and were hospitalized [18]. In a matched analysis of sotrovimab effectiveness, conducted using data from the US Department of Veterans Affairs, 3.6% (n = 92/2557) of sotrovimab-treated patients experienced 30-day COVID-19-related hospitalization or all-cause mortality during BA.1 predominance, compared with 7.1% (n = 735/10,297) of untreated patients [30]. A recent study of North-West London during BA.1, BA.2, and BA.5 predominance also showed higher rates of hospitalization for untreated patients than the current study, with 6.2% experiencing an all-cause hospitalization compared with 5.0% of sotrovimab-treated patients [31].

Data from the studies above indicate there has been an overall improvement in the proportion of patients experiencing severe COVID-19 outcomes over time and through different periods of subvariant predominance. This likely reflects the accrual of infection rates (and hence natural immunity), vaccine uptake, improved COVID-19 management, and reduced disease severity due to ongoing viral evolution. As mentioned previously, there is substantial variability in the proportion of untreated patients being hospitalized, ranging from > 5% [11, 20, 24] to 2.8% in the current study. Possible explanations for the differences in these rates are linked to the study time period, variant of concern, differences between administrative claims and EHR data, differences in geography (e.g., claims tend to be nationally representative vs EHR data from a health system in a specific region), differences in treatment policies, and differences in vaccination rates. Mayo Clinic also reports favorable COVID-19 outcomes compared with US national averages, likely reflecting the standardized, multidisciplinary, team-based, consensus-driven treatment approaches used [20, 23, 32].

Sotrovimab, other mAbs, and antiviral treatments were administered quickly after COVID-19 diagnosis (mean [SD] time to treatment was 1.8 [1.2], 2.0 [1.4], and 1.3 [0.7], respectively); this rapid time to treatment reflects the centralized approach where treatment is coordinated by a unified team. As anticipated, there was a distinct temporal trend in when therapies were utilized, which likely reflected the EUA status of the individual treatments. Despite receiving EUA in May 2021 [7], the first administration of sotrovimab in Mayo Clinic’s EHR did not occur until October 2021, with the majority of use occurring from December 2021–February 2022 when the Omicron BA.1 subvariant predominated [17]; the majority of utilization for the other mAbs occurred from August 2021–November 2021 when the Delta variant predominated [33]. This difference in utilization over a short time period reflects the shift in the variant of concern (with December 2021 representing the transition from Delta to Omicron BA.1 [17]) and the subsequent EUA status (e.g., the other mAbs were “deauthorized” in early 2022 [34]), as well as the differing impact on health systems and public policies. Furthermore, when initially made available, sotrovimab had to be purchased, whereas the government provided other mAbs free of charge.

Our results also indicate differences in baseline characteristics and comorbidities between study cohorts (e.g., the control cohort tended to be younger and healthier compared with the treated cohorts); this likely represented the strategy of providing treatment to the highest-risk groups, especially during periods when treatment supply was scarce and unable to keep up with the surge of cases during Delta and Omicron waves [25].

This study has several limitations. Firstly, as this is a retrospective observational study, caution should be exercised when interpreting results due to the potential of bias from residual or unmeasured confounding. Certain attributes that influence treatment choice and endpoints of interest may not be captured in Mayo Clinic’s EHR data, such as symptom and severity data. The possibility of residual confounding due to the absence of variant sequencing data also cannot be excluded. Certain comorbid conditions may have been omitted if not appropriately documented, especially for patients new to Mayo Clinic because of the COVID-19 pandemic and who did not have pre-existing documentation in the EHR. It should be noted that we identified a substantial number of sotrovimab-treated patients who did not have evidence of meeting the high-risk EUA criteria. Furthermore, this is a cohort of multimorbid patients with complex medical needs, and our reporting of all-cause outcomes likely includes patients hospitalized for monitoring or reasons unrelated to COVID-19, which may affect interpretation of results. The geography and demographics of Mayo Clinic’s patient referral base also means that these findings may not translate to other US populations with different demographic characteristics, comorbid medical conditions, and resources at their disposal. Data missingness is a well-known problem for EHR studies; some encounters may not be fully captured, especially when patients seek care at a different facility not affiliated with Mayo Clinic. This represents a limitation of real-world data and may differ across sites. However, these inconsistencies are expected to affect all cohorts equally. It should also be noted that this study may be liable to immortal time bias given the 10-day period for early treatment. Finally, the antiviral group had a low sample size due to the scarcity of supply during the study period.

## Conclusions

Formal comparison of PS-matched sotrovimab-treated and untreated high-risk patients showed no significant difference in the proportion of patients experiencing all-cause hospitalization or mortality. However, patients treated with sotrovimab had significantly fewer ICU admissions and required significantly less respiratory support compared with untreated controls.

## Supporting information

S1 File (DOCX)
