# Supplementary material for: Real-world effectiveness of sotrovimab in preventing hospitalization and mortality in high-risk patients with COVID-19 in the United States: A cohort study from the Mayo Clinic electronic health records
Source: PLoS One. 2024 Jul 16;19(7):e0304822. doi: 10.1371/journal.pone.0304822 (PMC11251586; doi:10.1371/journal.pone.0304822)
Supplement: S1 File — (DOCX) [file pone.0304822.s001.docx]

# Supplementary material

# Real-world effectiveness of sotrovimab in preventing hospitalization and mortality in high-risk patients with COVID-19 in the United States: A cohort study from the Mayo Clinic electronic health records

Christopher F. Bell,^1^ Daniel C. Gibbons,^2^ Myriam Drysdale,^2^ Helen J. Birch,^2^
Emily J. Lloyd,^2^ Vishal Patel,*^2^ Corinne Carpenter,*^3^ Katherine Carlson,^3^
Ediz S. Calay,^3^ Arjun Puranik,^3^ Tyler E. Wagner,^3^ John C. O’Horo,^4^ Raymund R. Razonable^4^

^1^GSK, Durham, North Carolina, USA
^2^GSK, Brentford, UK
^3^nference, Cambridge, Massachusetts, USA
^4^Mayo Clinic, Rochester, Minnesota, USA

*At the time of study.

**Corresponding author**

Christopher F. Bell

GSK, 410 Blackwell Street, Durham, North Carolina 27701, USA

Tel: 9195183852

Email*:* christopher.f.bell@gsk.com

## Supplementary Table 1. EUA criteria for being at high risk of developing severe COVID-19 [1]

| Criterion | Additional information |
| --- | --- |
| Aged ≥ 65 years | Derived from “age” |
| Obesity | Per ICD-10 diagnosis codes |
| Pregnancy |  |
| History of CKD (any stage) |  |
| History of CKD (stage ≥ 3) |  |
| History of type 1 diabetes |  |
| History of type 2 diabetes |  |
| Immunosuppressive disease |  |
| Immunosuppressive treatment | Defined as ≥ 2 drug exposure events for systemic corticosteroid therapy in the past 365 days or ≥ 1 systemic non-corticosteroid immunosuppressants drug exposures in 365 days |
| Cardiovascular disease  (including congenital heart disease) or hypertension | Per ICD-10 diagnosis codes |
| Chronic lung disease (COPD or asthma) |  |
| Sickle cell disease |  |
| Neurodevelopmental disorders |  |
| Medical-related technological dependence | The presence of a procedure occurrence or device exposure event relating to any of the following: respiratory aspirator, gastrostomy or jejunostomy, Mitrofanoff procedure, nasogastric tube, renal replacement therapy, total parenteral nutrition, or ventricular assistance |
| Liver disease | Per ICD-10 diagnosis codes |

CKD, chronic kidney disease; COPD, chronic obstructive pulmonary disease; COVID-19, coronavirus disease 2019; EUA, Emergency Use Authorization; ICD-10, International Classification of Disease version 10.

## Supplementary Table 2. Patient demographics pre- and post-PS-matching across the sotrovimab-treated and untreated (control) cohorts

| Attribute | | Before matching | | | | | After matching | | | | |
| --- | --- | --- | --- | --- | --- | --- | --- | --- | --- | --- | --- |
|  |  | Sotrovimab | | Control | | SMD | Sotrovimab | | Control | | SMD |
|  |  | N | % | N | % |  | N | % | N | % |  |
| Age (years) | < 55 | 478 | 34.92 | 14,287 | 51.96 | –0.35 | 324 | 37.94 | 658 | 38.52 | –0.01 |
|  | 55–64 | 233 | 17.02 | 4412 | 16.05 | 0.03 | 149 | 17.45 | 324 | 18.97 | –0.04 |
|  | 65–74 | 415 | 30.31 | 5307 | 19.30 | 0.26 | 269 | 31.50 | 491 | 28.75 | 0.06 |
|  | ≥ 75 | 243 | 17.75 | 3489 | 12.69 | 0.14 | 112 | 13.11 | 235 | 13.76 | –0.02 |
| Sex | Female | 794 | 58.00 | 15,965 | 58.07 | 0.00 | 526 | 61.59 | 1012 | 59.25 | 0.05 |
|  | Male | 575 | 42.00 | 11,529 | 41.93 | 0.00 | 328 | 38.41 | 696 | 40.75 | –0.05 |
|  | Unknown | 0 | 0.00 | 1 | 0.00 | –0.01 | 0 | 0.00 | 0 | 0.00 | - |
| Race/ethnicity | Asian | 18 | 1.31 | 528 | 1.92 | –0.05 | 10 | 1.17 | 16 | 0.94 | 0.02 |
|  | Black/African American | 47 | 3.43 | 1160 | 4.22 | –0.04 | 28 | 3.28 | 35 | 2.05 | 0.08 |
|  | Hispanic | 55 | 4.02 | 1575 | 5.73 | –0.08 | 27 | 3.16 | 32 | 1.87 | 0.08 |
|  | Native American | 8 | 0.58 | 157 | 0.57 | 0.00 | 5 | 0.59 | 8 | 0.47 | 0.02 |
|  | Native Hawaiian/ Pacific Islander | 2 | 0.15 | 54 | 0.20 | –0.01 | 1 | 0.12 | 1 | 0.06 | 0.02 |
|  | Other | 11 | 0.80 | 454 | 1.65 | –0.08 | 5 | 0.59 | 8 | 0.47 | 0.02 |
|  | Unknown | 10 | 0.73 | 239 | 0.87 | –0.02 | 9 | 1.05 | 4 | 0.23 | 0.10 |
|  | White/Caucasian | 1273 | 92.99 | 24,903 | 90.57 | 0.09 | 796 | 93.21 | 1636 | 95.78 | –0.11 |
| Body mass index (kg/m^2)^ | < 18.5 | 5 | 0.37 | 98 | 0.36 | 0.00 | 2 | 0.23 | 2 | 0.12 | 0.03 |
|  | 18.5 to < 25 | 127 | 9.28 | 2324 | 8.45 | 0.03 | 64 | 7.49 | 142 | 8.31 | –0.03 |
|  | 25 to < 30 | 288 | 21.04 | 4259 | 15.49 | 0.14 | 166 | 19.44 | 324 | 18.97 | 0.01 |
|  | 30 to < 35 | 340 | 24.84 | 8721 | 31.72 | –0.15 | 218 | 25.53 | 423 | 24.77 | 0.02 |
|  | 35 to < 40 | 265 | 19.36 | 5143 | 18.71 | 0.02 | 177 | 20.73 | 319 | 18.68 | 0.05 |
|  | ≥ 40 | 280 | 20.45 | 5532 | 20.12 | 0.01 | 188 | 22.01 | 409 | 23.95 | –0.05 |
|  | Unknown | 64 | 4.67 | 1418 | 5.16 | –0.02 | 39 | 4.57 | 89 | 5.21 | –0.03 |

PS, propensity score; SMD, standardized mean difference.

## Supplementary Table 3. Patient clinical characteristics pre- and post-PS-matching across the sotrovimab-treated and untreated (control) cohorts

| Attribute | Before matching | | | | | After matching | | | | |
| --- | --- | --- | --- | --- | --- | --- | --- | --- | --- | --- |
|  | Sotrovimab | | Control | | SMD | Sotrovimab | | Control | | SMD |
|  | N | % | N | % |  | N | % | N | % |  |
| Cancer | 174 | 12.71 | 1685 | 6.13 | 0.23 | 74 | 8.67 | 106 | 6.21 | 0.09 |
| Cardiovascular disease | 253 | 18.48 | 3233 | 11.76 | 0.19 | 96 | 11.24 | 222 | 13.00 | –0.05 |
| Cerebrovascular disease | 43 | 3.14 | 538 | 1.96 | 0.08 | 9 | 1.05 | 26 | 1.52 | –0.04 |
| Chronic lung disease | 90 | 6.57 | 1700 | 6.18 | 0.02 | 47 | 5.50 | 79 | 4.63 | 0.04 |
| Chronic pulmonary disease | 95 | 6.94 | 1825 | 6.64 | 0.01 | 51 | 5.97 | 83 | 4.86 | 0.05 |
| Congestive heart failure | 68 | 4.97 | 782 | 2.84 | 0.11 | 18 | 2.11 | 50 | 2.93 | –0.05 |
| Connective tissue disease | 68 | 4.97 | 426 | 1.55 | 0.19 | 17 | 1.99 | 39 | 2.28 | –0.02 |
| Dementia | 5 | 0.37 | 145 | 0.53 | –0.02 | 1 | 0.12 | 2 | 0.12 | 0.00 |
| HIV/AIDS | 1 | 0.07 | 12 | 0.04 | 0.01 | 0 | 0.00 | 3 | 0.18 | –0.06 |
| History of CKD (stage ≥ 3) | 50 | 3.65 | 448 | 1.63 | 0.13 | 18 | 2.11 | 27 | 1.58 | 0.04 |
| History of CKD (any stage) | 114 | 8.33 | 1202 | 4.37 | 0.16 | 45 | 5.27 | 91 | 5.33 | 0.00 |
| History of type 1 diabetes | 15 | 1.10 | 196 | 0.71 | 0.04 | 7 | 0.82 | 18 | 1.05 | –0.02 |
| History of type 2 diabetes | 177 | 12.93 | 2203 | 8.01 | 0.16 | 83 | 9.72 | 180 | 10.54 | –0.03 |
| Immunosuppressive treatment^a^ | 312 | 22.79 | 1709 | 6.22 | 0.48 | 136 | 15.93 | 150 | 8.78 | 0.22 |
| Immunosuppressive disease | 162 | 11.83 | 1749 | 6.36 | 0.19 | 68 | 7.96 | 158 | 9.25 | –0.05 |
| Received solid organ transplant | 213 | 15.56 | 1755 | 6.38 | 0.30 | 82 | 9.60 | 161 | 9.43 | 0.01 |
| Received SCT/BMT | 52 | 3.80 | 284 | 1.03 | 0.18 | 14 | 1.64 | 11 | 0.64 | 0.09 |
| Received anti-CD20 medication | 39 | 2.85 | 195 | 0.71 | 0.16 | 18 | 2.11 | 12 | 0.70 | 0.12 |
| Tier 1 immunocompromised | 281 | 20.53 | 2112 | 7.68 | 0.38 | 105 | 12.30 | 179 | 10.48 | 0.06 |
| Immunocompromised  (any definition) | 468 | 34.19 | 4171 | 15.17 | 0.45 | 190 | 22.25 | 377 | 22.07 | 0.00 |
| Liver disease | 60 | 4.38 | 715 | 2.60 | 0.10 | 29 | 3.40 | 49 | 2.87 | 0.03 |
| Medical-related  technological dependence^b^ | 0 | 0.00 | 3 | 0.01 | –0.01 | 0 | 0.00 | 0 | 0.00 | - |
| Metastatic carcinoma | 38 | 2.78 | 291 | 1.06 | 0.13 | 17 | 1.99 | 25 | 1.46 | 0.04 |
| Mild liver disease | 21 | 1.53 | 189 | 0.69 | 0.08 | 10 | 1.17 | 17 | 1.00 | 0.02 |
| Myocardial infarction | 12 | 0.88 | 192 | 0.70 | 0.02 | 5 | 0.59 | 10 | 0.59 | 0.00 |
| Neurodevelopmental disorder | 18 | 1.31 | 798 | 2.90 | –0.11 | 7 | 0.82 | 12 | 0.70 | 0.01 |
| Paraplegia and hemiplegia | 1 | 0.07 | 39 | 0.14 | –0.02 | 0 | 0.00 | 2 | 0.12 | –0.05 |
| Peptic ulcer disease | 4 | 0.29 | 83 | 0.30 | 0.00 | 2 | 0.23 | 3 | 0.18 | 0.01 |
| Peripheral vascular disease | 53 | 3.87 | 568 | 2.07 | 0.11 | 22 | 2.58 | 37 | 2.17 | 0.03 |
| Pregnancy | 159 | 11.61 | 2474 | 9.00 | 0.09 | 113 | 13.23 | 190 | 11.12 | 0.06 |
| Renal disease | 144 | 10.52 | 1453 | 5.28 | 0.19 | 51 | 5.97 | 116 | 6.79 | –0.03 |
| Sickle cell disease | 0 | 0.00 | 11 | 0.04 | –0.03 | 0 | 0.00 | 0 | 0.00 | - |

^a^Immunosuppressive treatment was defined as ≥ 2 drug exposure events for systemic corticosteroid therapy in the past 365 days or ≥ 1 systemic non-corticosteroid immunosuppressants drug exposures in the past 365 days. ^b^Medical-related technological dependence was defined as the presence of a procedure occurrence or device exposure event relating to any of the following: respiratory aspirator, gastro or jejunostomy, Mitrofanoff procedure, nasogastric tube, renal replacement therapy, total parenteral nutrition, or ventricular assistance.

AIDS, acquired immune deficiency syndrome; BMT, bone marrow transplant; CKD, chronic kidney disease; HIV, human immunodeficiency virus; PS, propensity score; SCT, stem cell transplant; SMD, standardized mean difference.

## Supplementary Table 4. Patient vaccination status and treatment month pre- and post-PS-matching across the sotrovimab-treated and untreated (control) cohorts

| Attribute | | Before matching | | | | | After matching | | | | |
| --- | --- | --- | --- | --- | --- | --- | --- | --- | --- | --- | --- |
|  |  | Sotrovimab | | Control | | SMD | Sotrovimab | | Control | | SMD |
|  |  | N | % | N | % |  | N | % | N | % |  |
| Vaccination status | No record | 356 | 26.00 | 11,638 | 42.33 | –0.35 | 251 | 29.39 | 502 | 29.39 | 0.00 |
|  | Partial | 68 | 4.97 | 1271 | 4.62 | 0.02 | 12 | 1.41 | 24 | 1.41 | 0.00 |
|  | Full | 435 | 31.78 | 9506 | 34.57 | –0.06 | 293 | 34.31 | 586 | 34.31 | 0.00 |
|  | Boosted | 510 | 37.25 | 5080 | 18.48 | 0.43 | 298 | 34.89 | 596 | 34.89 | 0.00 |
| Treatment month | May 2021 | 0 | 0.00 | 42 | 0.15 | –0.06 | 0 | 0.00 | 0 | 0.00 | - |
|  | June 2021 | 0 | 0.00 | 189 | 0.69 | –0.12 | 0 | 0.00 | 0 | 0.00 | - |
|  | July 2021 | 0 | 0.00 | 593 | 2.16 | –0.21 | 0 | 0.00 | 0 | 0.00 | - |
|  | August 2021 | 0 | 0.00 | 1516 | 5.51 | –0.34 | 0 | 0.00 | 0 | 0.00 | - |
|  | September 2021 | 0 | 0.00 | 1616 | 5.88 | –0.35 | 0 | 0.00 | 0 | 0.00 | - |
|  | October 2021 | 82 | 5.99 | 1662 | 6.04 | 0.00 | 50 | 5.85 | 94 | 5.50 | 0.02 |
|  | November 2021 | 192 | 14.02 | 2605 | 9.47 | 0.14 | 141 | 16.51 | 296 | 17.33 | –0.02 |
|  | December 2021 | 214 | 15.63 | 3459 | 12.58 | 0.09 | 145 | 16.98 | 183 | 10.71 | 0.18 |
|  | January 2022 | 505 | 36.89 | 12,251 | 44.56 | –0.16 | 338 | 39.58 | 805 | 47.13 | –0.15 |
|  | February 2022 | 264 | 19.28 | 2368 | 8.61 | 0.31 | 138 | 16.16 | 271 | 15.87 | 0.01 |
|  | March 2022 | 111 | 8.11 | 647 | 2.35 | 0.26 | 42 | 4.92 | 59 | 3.45 | 0.07 |
|  | April 2022 | 1 | 0.07 | 547 | 1.99 | –0.19 | 0 | 0.00 | 0 | 0.00 | - |
|  | May 2022 | 0 | 0.00 | 0 | 0.00 | - | 0 | 0.00 | 0 | 0.00 | - |

Patients were considered partially vaccinated if they had received one vaccination during the assessment period with an mRNA vaccine (Pfizer-BioNTech [BNT162b2] or Moderna [mRNA-1273]). Patients were considered fully vaccinated if they had received ≥ 2 vaccinations during the assessment period with an mRNA vaccine (Pfizer-BioNTech [BNT162b2] or Moderna [mRNA-1273]) or had received a single dose of the Johnson & Johnson viral vector vaccine
(JNJ-784336725). Patients were considered fully vaccinated with a booster if they had received ≥ 3 vaccinations with an mRNA vaccine, or ≥2 vaccinations ≥ 1 JNJ-784336725 vaccination,

mRNA, messenger ribonucleic acid; PS, propensity score; SMD, standardized mean difference.

# Supplementary reference

1. U.S. Food and Drug Administration. Fact sheet for healthcare providers: Emergency Use Authorization for sotrovimab [Internet]. 2023 March [cited 2023 November 15]. Available from: <https://www.fda.gov/media/149534/download>.
